# Agency and Communion From the Perspective of Self Versus Others: The Moderating Role of Social Class

**DOI:** 10.3389/fpsyg.2019.02867

**Published:** 2019-12-18

**Authors:** Xiaochen Chen, Muzi Li, Qingwang Wei

**Affiliations:** ^1^Department of Psychology, Renmin University of China, Beijing, China; ^2^Mental Health and Education Center, Minzu University of China, Beijing, China

**Keywords:** social class, communion, agency, self, other

## Abstract

Communion and agency are the two fundamental dimensions of social perception. The dual perspective model (DPM) predicts that communion is more desirable and important in the other perspective, whereas agency is more desirable and important in the self-perspective. Social class psychology has suggested that social class is also systematically linked to one’s orientation toward communion and agency. However, little is known about how basic perspectives (i.e., self versus other) and social class jointly affect the primacy of communion and agency in social cognition. The current study attempted to address this gap by asking participants from different social class conditions to rate the importance of communal and agentic traits both with respect to the self and to another person. Results indicated that lower class individuals rated communal traits as more important than agentic ones for others, whereas upper class individuals rated agentic traits as more important than communal ones for themselves. This work extends both DPM and the social class psychology. Current findings could provide important practical implications for inter-class communications.

## Introduction

Communion and agency are the fundamental dimensions (i.e., the Big Two) in social cognition, and the primacy of the Big Two is linked to the actor (self) versus observer (other) perspectives (Abele and Wojciszke, 2007, 2014). A distinct but related line of literature yielded from social class psychology has suggested that social class is also systematically linked to one’s orientation toward communion and agency (see Rucker et al., 2018 for a review). Yet, little is known about how basic perspectives (self versus other) and social class jointly affect the desirability of communion and agency. For example, are communal traits particularly important for lower class individuals when perceiving others? Are agentic characteristics more desirable for upper class individuals when evaluating themselves? Answers to such questions could provide important practical implications to promote healthy inter-class communications.

### Dual Perspective Model of Communion and Agency

A large body of research has shown that communion and agency are the fundamental dimensions of social cognition (e.g., Judd et al., 2005; Abele and Wojciszke, 2007, 2014; Fiske et al., 2007). Communion arises from striving to integrate the self in a larger social unit through caring for others and involves qualities such as benevolence, cooperativeness, and empathy. Agency arises from striving to individuate and expand the self and involves qualities such as efficiency, competence, and assertiveness (Abele and Wojciszke, 2014). Social groups have evolved over time and can now share resources, reduce risk, and help overcome stress or threat, which has made them indispensable for human beings. Therefore, there should be a selective advantage to possess communal traits necessary to build and maintain social relationships. This primacy of communion has been evident in language and information processing (e.g., Ybarra et al., 2008; Abele and Bruckmüller, 2011). The DPM further pointed out that the fundamental dimensions are differently linked to the basic perspectives in social cognition, that is the actor (self) versus the observer (other) perspectives. Communal traits can inform the perceivers about the “benevolent versus malevolent” intentions of the trait possessors, thus are other-profitable. Agentic traits, on the other hand, allow the trait possessors to effectively pursue their own goals, thus are self-profitable. According to this profitability principle, the DPM predicts that communion is more desirable and important in the other perspective; conversely, agency is more desirable and important in the self-perspective.

### Social Class and Cognitive Tendencies

The primacy of communion is not only moderated by perspectives but also moderated by one’s position. Previous research has shown that power could orient an individual toward greater agency as opposed to communion (Rucker et al., 2012; Cislak, 2013). Social class psychology has suggested that the effects of social class on people’s orientation toward communion and agency are similar to the effects of power (Rucker et al., 2018). Previous work on social status and intergroup differentiation has shown that high-status groups differentiated themselves positively on competence (agency), whereas low-status groups differentiated themselves positively on warmth (communion) (Oldmeadow and Fiske, 2010). Advantage in the form of higher social class (i.e., abundant resources and elevated rank) increases agency, whereas the disadvantage from lower social class (i.e., diminished resources and lower rank) increases communion (Kraus et al., 2012; Rucker et al., 2018). Empirical studies have revealed that lower class individuals were more accurate in the detection of others’ emotions (Kraus et al., 2010, 2011), more concerned with connections to the community (Eriksson and Simpson, 2014), and were more likely to help others (Piff et al., 2010). In contrast, upper class individuals were more concerned with their own achievements and uniqueness and showed more positive self-evaluations (Stephens et al., 2007; Kraus et al., 2012; Kraus and Park, 2014; Piff, 2014).

### The Current Study

Despite these findings, few studies have systematically examined potential social class differences in the relative importance of communion and agency from the self-perspectives versus the other perspectives, especially in the context of trait ratings. The current study attempts to fill this gap by asking participants from different social class conditions to rate the importance of a number of agentic and communal traits both with respect to the self and to another person. Based on the DPM (Abele and Wojciszke, 2007, 2014), we predicted that communal traits would be rated as more important than agentic ones (i.e., C over A) for others, whereas agentic traits would be rated as more important than communal traits (i.e., A over C) for the self. More importantly, we hypothesized that social class would moderate the link between basic perspectives and the importance of the Big Two. Because of the enhanced value of communion for the lower class, we expected that the C over A effect in the other-perspective would be more evident among lower-class individuals. In addition, since higher social class increases an agentic orientation, we expected that the C over A effect would be diminished or even completely offset among upper-class individuals. The same logic applies to trait importance ratings for the self. We expected that the A over C effect would be more evident among upper-class individuals due to enhanced agency yielded from class advantage, whereas this effect would be undermined or even offset among the lower class.

## Methods

### Participants and Design

Participants were 118 Chinese college students (30 men and 88 women; *M*_age_ = 22.0 years, SD = 1.75). The design was a 2 (dimension: communion versus agency) × 2 (perspective: self versus other) × 2 (social class: low versus high) mixed ANOVA, with repeated measures on the first two factors.

### Manipulation of Social Class

Participants took part in a manipulation of their relative social class, following the procedure developed by Piff et al. (2010). Participants were presented with an image of ladder with 10 rungs. They were instructed to think of the ladder “as representing where people stand in the Chinese society.” They were then randomly assigned to experience either high or low relative social class and received the following instructions:

Now, please compare yourself to the people at the very bottom (top) of the ladder. These are people who are the worst (best) off—those who have the least (most) money, least (most)education, and the least (most)respected jobs. In particular, we’d like you to think about how you are different from these people in terms of your family income, educational history, and job status. Where would you place yourself on this ladder relative to these people at the very bottom (top)?

To strengthen the manipulation, we instructed participants to write about a hypothetical interaction with a person from the bottom or top of the ladder; the bottom rung was coded as “1,” and the top rung was coded as “10.” Upper-class rank participants (*M* = 5.73, SD = 1.86), who compared themselves with people at the bottom of the latter, placed themselves significantly above lower-class rank participants (*M* = 4.69, SD = 1.50), who compared themselves with people at the top of the ladder, *t*(116) = 3.33, *p* < 0.01, *d* = 0.62. Thus, our manipulation successfully shifted participants’ perceptions of their subjective SES.

### Dependent Measures

To assess the importance of communion and agency, participants were asked to rate the importance of a number of positive traits (six communal: reliable, friendly, warm, generous, kind, considerate; six agentic: efficient, assertive, self-confident, diligent, persistent, competent) on a five-point scale ranging from 1 (*not important*) to 5 (*very important*) for the self and another person. All the traits were selected from Chinese Adjective Word System for Fundamental Dimensions of Social Cognition developed by Han and colleagues (Han et al., 2015).Reliability analyses showed that both the agency and the communion items formed internally consistent scales (communion: *α*’s = 0.68 and 0.73 for the self and others, respectively; agency: *α*’s = 0.74 and 0.81 for the self and others, respectively).We used averaged ratings as dependent measures. Half of the participants first answered the questions related to the self; the other half first answered the questions related to others. Preliminary analyses showed that the order of answering had no effect.

## Results

Trait importance ratings were subjected to a 2 (dimension: communion versus agency) × 2 (perspective: self versus other) × 2 (social class: low versus high) factorial ANOVA, with repeated measures on the first two factors. Results indicated that the main effect of dimension was significant, *F*(1,116) = 5.20, *p* < 0.05, $\eta_{p}^{2}$= 0.04. Communal traits (*M* = 4.14, SD = 0.51) were rated as more important than agentic traits (*M* = 4.01, SD = 0.54). The main effect of perspective was also significant, *F*(1, 116) = 35.32, *p* < 0.001, $\eta_{p}^{2}=$ 0.23. Traits for the self (*M* = 4.20, SD = 0.47) were rated as more important than those for others (*M* = 3.95, SD = 0.47). The main effect of social class was nonsignificant, *F*(1, 116) = 0.60, *p* = 0.44.

**Figure 1:**
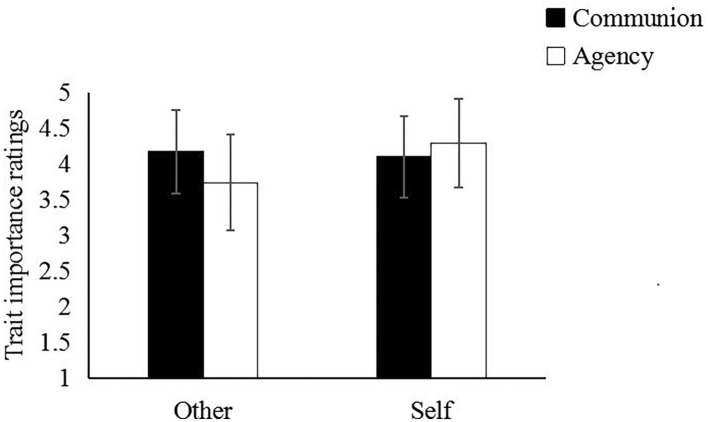
Importance of communal and agentic traits for the self versus for another person.

As hypothesized, the Dimension × Perspective interaction was significant, *F*(1,116) = 60.19, *p* < 0.001, $\eta_{p}^{2}$= 0.34. As shown in Figure 1, communal traits (*M* = 4.17, SD = 0.59) were rated as more important than agentic traits (*M* = 3.74, SD = 0.67) for others, *t*(117), *p* < 0.001, *d* = 0.52; whereas agentic traits (*M* = 4.29, SD = 0.62) were rated as more important than communal traits (*M* = 4.10, SD = 0.57) for the self, *t*(117), *p* < 0.01, *d* = 0.26. Communal traits were rated as equally important for others (*M* = 4.17, SD = 0.59) as for the self (*M* = 4.10, SD = 0.57), *t*(117) = 1.30, *p* = 0.19, *d* = 0.12. In contrast, agentic traits were rated as more important for the self (*M* = 4.29, SD = 0.62) than for others (*M* = 3.74, SD = 0.67), *t*(117) = 8.61, *p* < 0.001, *d* = 0.79. The Dimension × Social Class interaction was also significant, *F*(1, 116) = 26.60, *p* < 0.001, $\eta_{p}^{2}=$ 0.19. Lower-class participants rated communal traits (*M* = 4.25, SD = 0.47) as more important than agentic traits (*M* = 3.84, SD = 0.52), *t*(58) = 4.79, *p* < 0.001, *d* = 0.62; whereas upper-class participants rated agentic traits (*M* = 4.18, SD = 0.51) as more important than communal ones (*M* = 4.03, SD = 0.52), *t*(58) = 2.29, *p* < 0.05, *d* = 0.30. Put it differently, communal traits were rated as more important in the lower-class condition than in the upper-class condition, *t*(116) = 2.42, *p* < 0.05, *d* = 0.45, and vice versa for agentic traits, *t*(116) = 3.60, *p* < 0.001, *d* = 0.66. The Social Class × Perspective interaction was nonsignificant, *F*(1, 116) = 2.61, *p* = 0.11.

**Figure 2:**
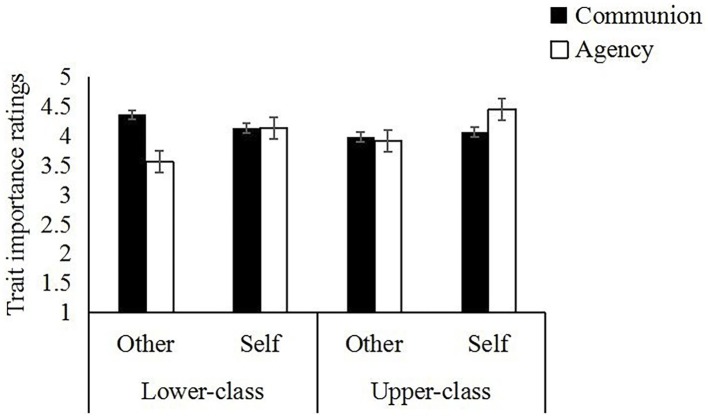
Ratings on importance of communion and agency by lower- versus upper-class individuals.

More pertinent to our concern, the Dimension × Perspective × Social Class interaction was also significant, *F*(1,116) = 4.72, *p* < 0.05, $\eta_{p}^{2}$ = 0.04. To further explore this critical three-way interaction, a 2 (dimension) × 2 (perspective) ANOVA was conducted within each social class condition. As shown in the left panel of Figure 2, for lower-class participants, the Dimension × Perspective interaction was significant, *F*(1, 58) = 38.33, *p* < 0.001, $\eta_{p}^{2}$ = 0.40. Communal traits (*M* = 4.36, SD = 0.51) were rated as significantly more important than agentic traits (*M* = 3.36, SD = 0.71) for others, *t*(58) = 7.20, *p* < 0.001, *d* = 0.94, whereas there was no difference in importance of communion (*M* = 4.14, SD = 0.54) and agency (*M* = 4.13, SD = 0.58) for the self, *t*(58) = 0.06, ns. Comparing ratings from different perspectives, results showed that lower-class participants rated communal traits as more important for others (*M* = 4.36, SD = 0.51) than for the self (*M* = 4.14, SD = 0.54), *t*(58) = 3.67, *p* < 0.01, *d* = 0.48; and the reversed pattern was found for agentic traits (*M*’s = 4.13, 3.36 for self- and other-perspectives, respectively), *t*(58) = 5.59, *p* < 0.001, *d* = 0.73. In contrast, for upper-class participants, the Dimension × Perspective interaction was also significant, *F*(1, 58) = 21.86, *p* < 0.001, $\eta_{p}^{2}$ = 0.27. As shown in the right panel of Figure 2, agentic traits (*M* = 4.45, SD = 0.61) were rated as significantly more important than communal traits (*M* = 4.07, SD = 0.60) for the self, *t*(58) = 4.54, *p* < 0.001, *d* = 0.59, whereas there was no difference in importance ratings of communion (*M* = 3.98, SD = 0.60) and agency (*M* = 3.91, SD = 0.56) for others, *t*(58) = 0.81, ns. Compared from different perspectives, results indicated that upper-class participants rated agentic traits as more important for the self (*M* = 4.45, SD = 0.61) than for others (*M* = 3.91, SD = 0.56), *t*(58) = 6.82, *p* < 0.001, *d* = 0.89; whereas ratings on communal traits did not differ significantly by perspectives, *t*(58) = 1.13, ns. To summarize, the C over A effect from the other-perspective was more evident among the lower-class individuals, and it was absent among the upper-class. In contrast, the A over C effect from the self-perspective was more evident among the upper-class individuals.

## Discussion

The current study contributes to the social cognition literature in several ways. First, it extends the DPM by integrating social class as a moderator. According to the DPM (Abele and Wojciszke, 2007, 2014), in the other-perspective, communion is more relevant and more important, whereas in the self-perspective, agency is more relevant and more important. Although the key tenets of the DPM have been largely supported, the relative importance of the Big Two within one perspective (i.e., within the perspective of the self or the perspective of another person) was inconsistent in empirical studies (e.g., Abele and Wojciszke, 2007; Wojciszke and Abele, 2008; Abele and Brack, 2013; Bi et al., 2013; Abele et al., 2014; Wei et al., 2018). In addition, due to its absolute adaptive value and the enhanced importance of communion from the other perspective, findings on the relative importance of communion from the self- versus the other-perspective was also inconclusive (e.g., Abele and Wojciszke, 2007, 2014). The DPM failed to provide a theoretically sound explanation for the mixed findings. The current study attempted to address this gap by integrating social class into the picture. Results indicated that the linkage between the fundamental dimensions and the basic perspectives varies as a function of people’s social class. Specifically, the C over A effect from the other-perspective was only found among the lower class, and it was only the lower-class participants who rated communal traits as more important for others (i.e., from the other-perspective) than for themselves (i.e., from the self-perspective). It is plausible that the lower social class context promotes a focus on others’ communal traits (Stephens et al., 2014; Rucker et al., 2018), which in turn magnifies the association between the other-perspective and a communion orientation among lower-class people. In contrast, although enhanced importance of agency from the self-perspective (compared to the other-perspective) was documented among both social classes, the A over C effect within the self-perspective was only found among the upper class, whose class context increases agency (Rucker et al., 2018).

Existing literature has documented the similarities in psychology of various forms of inequality. The advantaged (e.g., the upper class, people have more power) received more resources, opportunities, positive appraisals, and deference than do the disadvantaged (e.g., the lower class, people have less power) (Rucker et al., 2018). However, there are some differences between social class and power (Kraus and Stephens, 2012). For example, power tends to be less consistent and more specific to a given context or relationship (e.g., an employer-employee relation). Previous study has shown that the self-other outcome dependency increases importance of another person’s agency (Abele and Wojciszke, 2007). People who have more power tend to treat those with less power in an instrumental way to pursuit their own goals (Gruenfeld et al., 2008). Thus, it is quite plausible that the implicit instrumental association in a power context leads to enhanced focus on others’ agency among people with higher power (Cislak, 2013). In contrast, the experiences associated with social class are relatively stable and relationship independent. Our findings, unlike those yielded from the power research, showed no difference in upper-class individuals’ importance ratings of communion and agency for (unrelated) others. The current study therefore added to the nuanced associations between various forms of inequality and the DPM.

The current study also extends past research on social class psychology. Previous research has primarily focused on the level of communion and agency demonstrated by different social classes (e.g., Kraus et al., 2010; Piff et al., 2010). The present work calls attention to the need to consider the self-other distinction when examining social class disparities in cognitive tendencies. Our findings showed that it was only in the perspective of another person that lower-class individuals valued communion more than agency; and it was only from the perspective of the self that upper-class individuals valued agency more than communion. These findings have important practical implications to understand and reduce inter-group tension between people from different social class backgrounds. It could be inferred that in cross-class interactions, lower-class individuals would tend to care more about the communion of the upper class, however, upper-class individuals were inclined to focus on their own competence and were less concerned about their lack of warmth (e.g., Dubois et al., 2015). This mismatch on desired (communion versus agency) dimensions is a plausible source of dysfunctional inter-class interactions. Given the compensatory relation between competence and warmth in stereotype content (Fiske et al., 2002, 2015), emphasizing warmth and downplaying competence may be an effective impression management strategy for upper-class individuals in cross-class communications. For the lower class, downplaying warmth, as well as improving and demonstrating competence could be helpful to gain respect from the upper class.

Although we believe the current study makes significant contributions to the social cognition literature, there are a number of limitations and future avenues of research needed. First, the current study did not systematically examine and control the social desirability of communal and agentic traits, which might affect the results. Other research paradigm (e.g., open-ended questions) should be used in future research to cross-validate the current findings. Second, because participants in our study were Chinese college students in mainland China, we do not know whether the present findings hold across other cultural contexts. There is evidence suggesting that culture could shape the psychological correlations of social class (Na et al., 2016; Miyamoto et al., 2018). It is plausible that the scarcity of resources coupled with the cultural emphasis on interdependence lead to enhanced focus on others’ communion among lower-class individuals in China. However, this C over A effect might be reduced or offset in an independent cultural context, where the culture emphasis is on exerting one’s talents and personal goal attainment. It would be informative for future cross-culture research to explore whether the current findings could be generalized to independent cultural contexts. Future studies should also consider the mechanisms underlying social class differences in social perceptions of the Big Two. Due to the close linkage between social class contexts and independent-interdependent self-construal (Stephens and Townsend, 2013; Stephens et al., 2014), and the parallels between independence versus interdependence and agency versus communion (Wojciszke, 1997), it might be fruitful in follow-up studies to examine self-construal as a mediator of social class differences in preference for agentic versus communal traits.

## Data Availability Statement

The datasets generated for this study are available on request to the corresponding author.

## Ethics Statement

The studies involving human participants were reviewed and approved by the ethics committee of the Department of Psychology, Renmin University of China. The patients/participants provided their written informed consent to participate in this study.

## Author Contributions

All authors developed the conceptual background and designed the study. XC wrote the paper while the other authors provided comments. ML collected and analyzed the data, and both QW and XC assisted with data analyses. All authors approved the final version of the manuscript for submission.

### Conflict of Interest

The authors declare that the research was conducted in the absence of any commercial or financial relationships that could be construed as a potential conflict of interest.
